# Long-Term Effect of Non-Selective Beta-Blockers in Patients With Rheumatoid Arthritis After Myocardial Infarction—A Nationwide Cohort Study

**DOI:** 10.3389/fphar.2021.726044

**Published:** 2021-09-21

**Authors:** Sheng-Fu Liu, Chih-Kuo Lee, Kuan-Chih Huang, Lian-Yu Lin, Mu-Yang Hsieh, Ting-Tse Lin

**Affiliations:** ^1^ Department of Internal Medicine, National Taiwan University Hospital, Hsin-chu, Taiwan; ^2^ College of Medicine, National Taiwan University, Taipei, Taiwan; ^3^ Graduate Institute of Clinical Medicine, College of Medicine, National Taiwan University, Taipei, Taiwan; ^4^ Department of Internal Medicine, National Taiwan University Hospital, Taipei, Taiwan; ^5^ Institute of Biological Science and Technology, National Yang Ming Chiao Tung University, Hsin-Chu, Taiwan

**Keywords:** beta-blockers, myocardial infarction, rheumatoid arthiritis, mortality, prognosis

## Abstract

**Objectives:** Rheumatoid arthritis (RA) is an independent nontraditional risk factor for incidence of myocardial infarction (MI) and post-MI outcome is impaired in the RA population. Use of beta-blockers improves the long-term survival after MI in the general population while the protective effect of beta-blockers in RA patients is not clear. We investigate the impact of beta-blockers on the long-term outcome of MI among RA patients.

**Methods:** We identified RA subjects from the registries for catastrophic illness and myocardial infarction from 2003 to 2013. The enrolled subjects were divided into three groups according to the prescription of beta-blockers (non-user, non-selective, and β1-selective beta-blockers). The primary endpoint was all-cause mortality. We adjusted clinical variables and utilized propensity scores to balance confounding bias. Cox proportional hazards regression models were used to estimate the incidence of mortality in different groups.

**Results:** A total of 1,292 RA patients with myocardial infarction were enrolled, where 424 (32.8%), 281 (21.7%), and 587 (45.5%) subjects used non-user, non-selective, and β1-selective beta-blockers, respectively. Use of beta-blockers was associated with lower risk of all-cause mortality after adjustment with comorbidities, medications (adjusted hazard ratio [HR] 0.871; 95% confidence interval [CI] 0.727–0.978), and propensity score (HR 0.882; 95% CI 0.724–0.982). Compared with *β*1-selective beta-blockers, treatment with non-selective beta-blockers (HR 0.856; 95% CI 0.702–0.984) was significantly related to lower risk of mortality. The protective effect of non-selective beta-blockers remained in different subgroups including sex and different anti-inflammatory drugs.

**Conclusion:** Use of beta-blockers improved prognosis in post-MI patients with RA. Treatment with non-selective beta-blockers was significantly associated with reduced risk of mortality in RA patients after MI rather than *β*1-selective beta-blockers.

## Highlights

Treatment with beta-blockers improves long-term survival after myocardial infarction (MI) in patients with rheumatoid arthritis (RA). Non-selective beta-blockers, rather than β1-selective beta-blockers, significantly reduces risk of mortality in our analysis. The protective effect remained in the subgroups including sex and use of DMARDs, steroids, and statins. The possible mechanism of better protection in non-selective beta-blockers is an anti-inflammation effect, particular in the RA population.

## Introduction

Rheumatoid arthritis (RA) is the most common inflammatory arthritis with symmetric polyarticular involvement (Firestein, 2003). Not only causing disability of joints, patients with RA also have extra-articular involvement. In respect to cardiovascular disease, studies have established rheumatoid arthritis as an independent nontraditional risk factor, especially for myocardial infarction (MI), heart failure, and sudden cardiac death (Solomon et al., 2003; Solomon et al., 2006). The increased prevalence of coronary artery disease and MI among patients with RA is well documented, but the outcomes after MI seem not as good as in the general population. Among post-MI patients, those with concomitant RA had poor prognosis and increased risk of mortality, which was proportional to RA duration and steroid dosage (Palomäki et al., 2021). People who have had RA for at least 10 years have around a three-fold higher risk for myocardial infarction compared with the general population (Sattar and Mcinnes, 2005). Likewise, cardiovascular mortality was 50% higher in RA patients than in the general population, and ischemia heart disease (IHD) increased mortality risks by 59%, compared with non-RA people (Avina-Zubieta et al., 2008). These findings implied that RA disease activity and systemic inflammation are key elements underlying increased atherosclerotic burden and premature atherosclerosis (Choy et al., 2014; Sparks, 2019). The potential mechanism of MI in RA patients is that acute phase reactants drive synovial inflammation which raising circulating cytokines, like interleukin-6 or leptin, leading to a spectrum of proatherogenic changes and endothelial dysfunction and damage (Sattar and Mcinnes, 2005).

Beta-blockers are an important medication for improving patients’ long-term survival after myocardial infarction. In the era of percutaneous coronary intervention, several prospective cohort studies and current guidelines have also indicated that treatment with beta-blockers is associated with reduced mortality in patients suffering from acute MI (Nakatani et al., 2013; Amsterdam et al., 2014; Choo et al., 2014; Ibanez et al., 2018; Collet et al., 2021). While there is considerable variation in the type of beta-blocker, most physicians assume that all beta-blockers exert a class effect to treat MI (Furberg et al., 1999; Lin et al., 2015). Benefits of beta-blockers for patients with MI include anti-ischemic, antihypertensive, antiarrhythmic, and antithrombotic effects (Lopez-Sendon et al., 2004). Furthermore, some studies demonstrated antinociceptive and anti-inflammatory effects of beta-blockers (Martin et al., 2015; Valdes et al., 2017; Lin et al., 2020c). In particular, non-selective beta-blockers, such as propranolol, could reduce inflammatory cytokines including IL-6 and TNFα, and inflammation-related transcription factors such as NFκB and STAT3 (Haldar et al., 2018).

The protective effect of beta-blockers in post-MI patients with concomitant RA is not clear. In this nationwide study, we investigated the effect of beta-blockers on long-term outcome of MI among RA patients. We also explored the different impact between non-selective and β1-selective beta-blockers on long-term outcome.

## Methods

### Study Cohort

**FIGURE 1:**
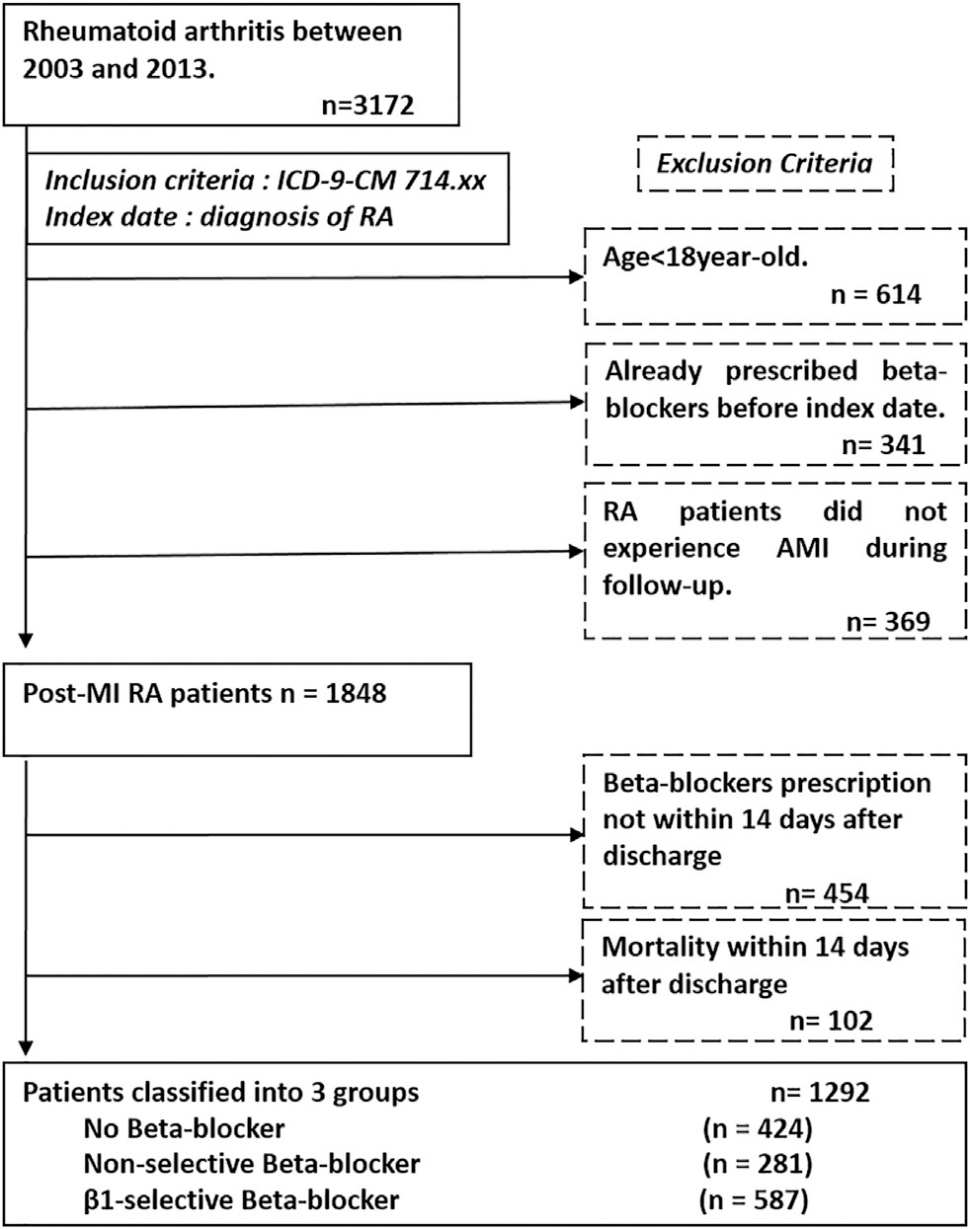
Patient flow diagram. We enrolled patients from the National Health Insurance database and identified subjects with diagnosis of rheumatoid arthritis (ICD-9-CM 714.xx) from 2003 to 2013. The index date of our study was defined as the date of diagnosis of rheumatoid arthritis. The exclusion criteria are listed in the dash line box, which are age <18 years, already prescribed beta-blockers before index date, not experienced myocardial infarction during follow-up, without beta-blockers prescription and incidence of death within 14 days after discharge from hospitalization of myocardial infarction.; Abbreviation: ICD-9-CM, International Classification of Diseases Ninth Revision Clinical Modification.

Like our previous studies, we used integrated medical and pharmacy claims data from the National Health Insurance Research Database (NHIRD) in Taiwan (Lin et al., 2017; Lin et al., 2019; Lin et al., 2020b). The NHIRD contains a nearly complete claims history of diagnosis and procedures, provided as the International Classification of Diseases Ninth Revision Clinical Modification (ICD-9-CM) codes, and drug dispensing for every beneficiary. Due to severe illness and heavy economic burden, patients with RA are registered and listed in the “Catastrophic Illnesses” system to waive almost all medical fees. All the medications, procedures, every OPD visit, and hospital admission covered by insurance are recorded in the database. Routine validations of the diagnoses by reviewing the original medical charts of all of the patients who applied for catastrophic illness registration are performed by the Bureau of National Health Insurance. We identified RA subjects through the use of the International Classification of Disease, Ninth Revision, Clinical Modification (ICD-9-CM) code 714.xx (excluding 714.3) in the catastrophic illness file from 2003 to 2013. We excluded patients younger than 18 years, previously prescribed with beta-blockers, or those with a diagnosis of myocardial infarction before RA. The identification of myocardial infarction was based on ICD9-CM codes (410.1–410.9) in the discharge diagnoses file. The index date for the study cohort was identified as the date of the first time diagnosis of RA. The enrolled subjects were divided into three groups according to the prescription of beta-blockers as follows: 1) patients not receiving beta-blockers treatment; 2) those receiving non-selective beta-blockers treatment, and 3) those receiving β1-selective beta-blockers treatment. To ensure the prescription of beta-blockers of RA patients after MI, those who received a prescription of beta-blockers more than 14 days after discharge and those who died within 14 days after discharge were excluded. A flowchart of the process used to identify study subjects is presented in Figure 1. The protocol for this study was approved by the Institutional Review Board of National Taiwan University Hospital and all participants gave written informed consent.

### Drug Use, Covariates, and Outcome

Beta-blockers users were defined as those taking these medications for more than 28 days during the follow-up period. In Taiwan, we utilize 12 kinds of beta-blockers (around 90 generic drugs with different doses). The majority of the treatment frequencies for beta-blockers were once daily (qd; 20–30%) and twice a day (bid; 70–80%). The non-selective beta-blockers included pindolol, alprenolol, nadolol, carvedilol, labetalol, and propranolol. The β1-selective beta-blockers included atenolol, acebutolol, bisoprolol and metoprolol, betaxolol, and nebivolol (Manrique et al., 2009). In terms of comorbidities, diabetes (250.X, 249.X), dyslipidemia (272.X), ischemic stroke (ICD9-CM code, 434.X), coronary artery disease (ICD9-CM code, 411.X-414.X, V17.3, V81.0), heart failure hospitalization (ICD9-CM code, 428.0–428.3, 429.9), or peripheral artery disease (ICD9-CM code, 250.7, 443.X, 444.2) were recorded within the last 12 months prior to the index date. Medications including angiotensin-converting enzyme inhibitors (ACEIs), angiotensin II receptor blockers (ARBs), calcium channel blockers (CCB), disease modifying anti-rheumatic drugs (DMARDs), steroids, non-steroid anti-inflammation drugs (NSAIDs), and statins were identified. The DMARDs included methotrexate, leflunomide, hydroxychloroquine, and sulfasalazine. The aim of this study was to investigate the protective effect of beta-blockers on post-MI patients with rheumatoid arthritis during long-term follow-up. The clinical outcome was all-cause mortality.

### Statistical Analyses

We used one-way ANOVA for continuous variables and the chi-square test for categorical variables to compare the baseline characteristics among four groups. Crude incidence rates for each event group with 95% confidence intervals (CI) were calculated from the total person-time exposure. The unadjusted rate ratios (RR) were calculated and examined using both univariate (Mantel–Haenszel) methods (Table 1). Owing to the heterogeneity of the three groups, we utilized multivariate Cox proportional hazard (PH) regression analyses to derive the adjusted hazard ratios (HRs) for incidence of mortality in different groups. Univariate Cox regression was used to determine crude HR in model 1. To eliminate bias and the effect of confounders, model 2 was adjusted for all confounders (age, gender, comorbidities, and medication usage) as main results. Furthermore, to balance the differences among the three groups, the propensity scores were constructed using multinomial logistic regression to model the receipt of non-selective or β1-selective beta-blockers as a function of baseline patient characteristics (Imbens, 2000). The covariates in this multinomial logistic regression model included all the background characteristics listed in Table 2 including age, gender, comorbidities, and medications. Propensity score-based adjustment was conducted to remove the initial bias. The procedure included a combination of propensity scores with other covariates in a regression model (model 3 in Table 3). Furthermore, it is possible that unrecognized residual confounders may impact the results, although propensity score adjustment is one of the strongest methods to control confounding factors. Therefore, we utilized the PH assumption testing methods, testing the correlation of scaled Schoenfeld residuals with time, to make sure that our PH assumption was met (Grambsch and Therneau, 1994). We used the Kaplan–Meier method to illustrate the event-free survival curves of the four groups. The log-rank test was applied to test the differences in survival among groups. To test the consistency of the results, we also did subgroup analyses for different sexes and use of statins, DMARDs, and steroids with adjustment for all confounders. For all HRs, we calculated 95% CIs. All *p* values were two-sided and a *p* value < 0.05 was considered statistically significant. All of the analyses were performed using the Statistical Package for the Social Sciences (SPSS) for Windows, Version 22 (SPSS, Inc., Chicago, IL), and SAS 9.4 software (SAS Institute Inc., Cary, NC).

**TABLE 1:** Incidence of mortality by prescriptions.

	Incidence of mortality			
	Total	Control (no use of beta-blockers)	Non-selective beta-blockers	β1-selective beta-blockers
Number of patients	1,292	424	281	587
Duration of follow-up	1,138 (432, 2,312)	1,194 (310, 2,741)	1,230 (393, 2,666)	1,079 (347, 2,029)
Median (IQR), days				
Mean (SD), days	1,521 (1,431)	1,690 (1,624)	1,621 (1,495)	1,351 (1,218)
Incident cases - *n* (%)	308 (23.8)	115 (27.1)	58 (20.6)	135 (22.9)
Incidence rate per 100 patient-years (95% CI)	5.71 (3.12–7.42)	5.85 (3.32–7.96)	4.64 (2.81–6.76)	5.79 (2.97–8.04)
Rate ratio (95% CI)		1.00	0.79 (0.56–0.97)*^a^	0.98 (0.61–1.13)

a *p* < 0.05. Abbreviations: AF, atrial fibrillation; IQR, interquartile range; SD, standard deviation.

**TABLE 2:** Patient baseline characteristics stratified by prescription of beta-blockers before and after propensity matching.

Variables	Control (no use of beta-blockers)	Non-selective beta-blockers	β1-selective beta-blockers
*N*	424	281	587
Age (mean, yrs)	68.1 ± 11.07	68.5 ± 10.49	68.8 ± 10.34
Gender, female %	238 (56.1)	208 (74)^a^	406 (69.2)^a^
HTN, %	291 (68.6)	231 (82.2)^a^	546 (93.0)^a^ ^,^ ^b^
DM, %	124 (29.2)	102 (36.3)^a^	273 (46.5)^a^ ^,^ ^b^
Dyslipidemia	163 (38.4)	119 (42.3)	292 (49.7)^a^ ^,^ ^b^
Ischemic stroke/TIA, %	55 (13.0)	39 (13.9)	99 (16.9)
CAD, %	285 (67.2)	215 (76.5)^a^	456 (77.7)^a^
PAD, %	86 (22.6)	101 (35.9)^a^	158 (26.9)^b^
CHF hospitalization, %	128 (30.2)	108 (38.4)^a^	232 (39.5)^a^
PCI at index hospitalization	365 (86.0)	243 (86.4)	513 (87.3)
CABG at index hospitalization	21 (4.9)	11 (3.9)^a^	19 (3.2)^a^
RA duration	2,824 ± 1,533	2,680 ± 1,575	2,890 ± 1,758
Medications			
ACEI/ARB, %	315 (74.2)	169 (60.1)^a^	456 (77.6)^b^
CCBs, %	189 (44.5)	126 (44.8)	287 (49.2)
DMARDs, %	121 (28.5)	75 (26.7)	201 (34.2)
NSAIDs, %	404 (95.3)	270 (96.1)	570 (97.1)
Steroids, %	332 (78.3)	214 (76.2)	452 (77.0)
Statins, %	165 (38.9)	115 (40.9)	369 (62.8)^a^ ^,^ ^b^

Abbreviations: ACEI, angiotensin converting enzyme inhibitor; ARB, angiotensin receptor blocker; CAD, coronary artery disease; CABG, coronary arteries bypass grafting surgery; CCBs, calcium channel blocker; CHF, congestive heart failure; DM, diabetes mellitus; DMARDs, disease modifying anti-rheumatic drugs; HTN, hypertension; NSAIDs, non-steroid anti-inflammation drugs; TIA, transient ischemic accident; PAD, peripheral artery disease; PCI, percutaneous coronary intervention.

a *p* < 0.05 compared with the no beta-blocker group.

b *p* < 0.05 compared with the group treated with non-selective beta-blockers.

**TABLE 3:** Hazard ratios (95% CI) of incident mortality in patients taking beta-blockers, with no beta-blocker treatment as the reference group.

	Model 1^a^		Model 2^b^		Model 3^c^	
	HR	95% CI	HR	95% CI	HR	95% CI
Beta-blockers vs control						
Incidence of mortality post MI	0.841^d^	0.668–0.952	0.871^d^	0.727–0.978	0.882^d^	0.724–0.982
Non-selective beta-blockers vs control						
Incidence of mortality post MI	0.822^d^	0.638–0.915	0.859^d^	0.587–0.992	0.876^d^	0.760–0.975
β1-selective beta-blockers vs control						
Incidence of mortality post MI	0.879^d^	0.650–0.994	0.897	0.694–1.152	0.891	0.710–1.154
Non-selective beta-blockers vs β1-selective beta-blockers						
Incidence of mortality post MI	0.884^d^	0.712–0.996	0.856^d^	0.702–0.984	0.872^d^	0.723–0.991

Abbreviations: CI, confidence interval; HR, hazard ratio; MI, myocardial infarction.

a Model 1: crude incidence.

b Model 2: adjusted for age, gender, risk factors (hypertension, diabetes mellitus, and dyslipidemia), comorbidities (stroke/transient ischemic accident, coronary artery disease, peripheral artery disease, heart failure), and medication usage (ACEI/ARB, CCBs, DMARDs, NSAIDs, steroids, and statins).

c Model 3: adjusted all variables in model 2 plus propensity score adjustment.

d *p* value <0.05.

## Results

### Patient Characteristics

There were 1,292 RA patients who met the study inclusion criteria; 424 (32.8%) did not use beta-blockers while 281 (21.7%) used non-selective beta-blockers and 587 (45.5%) used β1-selective beta-blockers. Among the non-selective beta-blockers group, 1.4, 0.7, 1.7, 11, 8.5, and 76.7% of patients were prescribed with pindolol, alprenolol, nadolol, carvedilol, labetalol, and propranolol, respectively. In respect to the β1-selective beta-blockers group, 32.4, 6.6, 31.2, 8.1, 1.2, and 20.5% of patients were prescribed with atenolol, acebutolol, bisoprolol and metoprolol, betaxolol, and nebivolol, respectively. Patients not receiving beta-blocker treatment served as the control group. The median follow-up time was 1,138 days (25th–75th IQR, 432 and 2,312 days). The algorithm is listed in Figure 1.

Clinical and demographic characteristics are listed in Table 2. Overall, patients without beta-blocker treatment were at a similar age to those with beta-blockers. There were significantly fewer female patients in the non-beta-blocker group. The duration from RA diagnosis to incident ACS was around 7–8 years (2,800 days) and there was no significant difference among the three groups. The prevalence of risk factors including HTN, DM, and dyslipidemia were higher in the beta-blocker group as well. The prevalence of comorbidities including CAD, PAD, and HF hospitalization was also higher in the beta-blocker group than in the control group. Between subjects taking non-selective and β1-selective beta-blockers, HTN (82.2 vs 93.0%), DM (36.3 vs 46.5%), and dyslipidemia (42.3 vs 49.7%) were more prevalent in those taking β1-selective beta-blockers. On the contrary, PAD (35.9 vs 26.9%) was more common in those taking non-selective beta-blockers. In respect to revascularization strategies, the proportion of percutaneous coronary intervention (PCI) was around 85% among the three groups, and coronary artery bypass grafting surgery was significantly higher in the control group. Among medication use, the prescription of ACEI/ARB was more common in the control group and β1-selective beta-blockers group. The use of calcium channel blockers (CCBs) was similar among the three groups. Of note, there was no significant difference among the three groups in respect to treatment with DMARDs, NSAIDs, and steroids. At last, the prescription of statins was more common in the β1-selective beta-blocker group.

### Main Outcome: All-Cause Mortality

The median durations of follow-up were 1,194, 1,230, and 1,079 days in the control, non-selective, and β1-selective beta-blocker groups, respectively. Overall, the incidence rate of all-cause mortality was 5.71 per 100 patient-years. As demonstrated in Table 1, the overall all-cause mortality rate during the entire follow-up period was numerically less in the non-selective and β1-selective beta-blockers groups (20.6 and 22.9%) and higher in the control group (27.1%) (Table 1). Table 1 also shows the crude incidence rate which was numerically higher in the control group (5.85 per 100 patient-years) and β1-selective beta-blockers group (5.79 per 100 patient-years) and lower in the non-selective beta-blocker group (4.64 per 100 patient-years). There was a significantly reduced rate ratio for the non-selective beta-blockers group when compared with control (RR 0.79, 95% CI 0.56–0.97, *p* < 0.05) while RR was not below statistical significance for users of β1-selective beta-blockers (Table 1).

**FIGURE 2:**
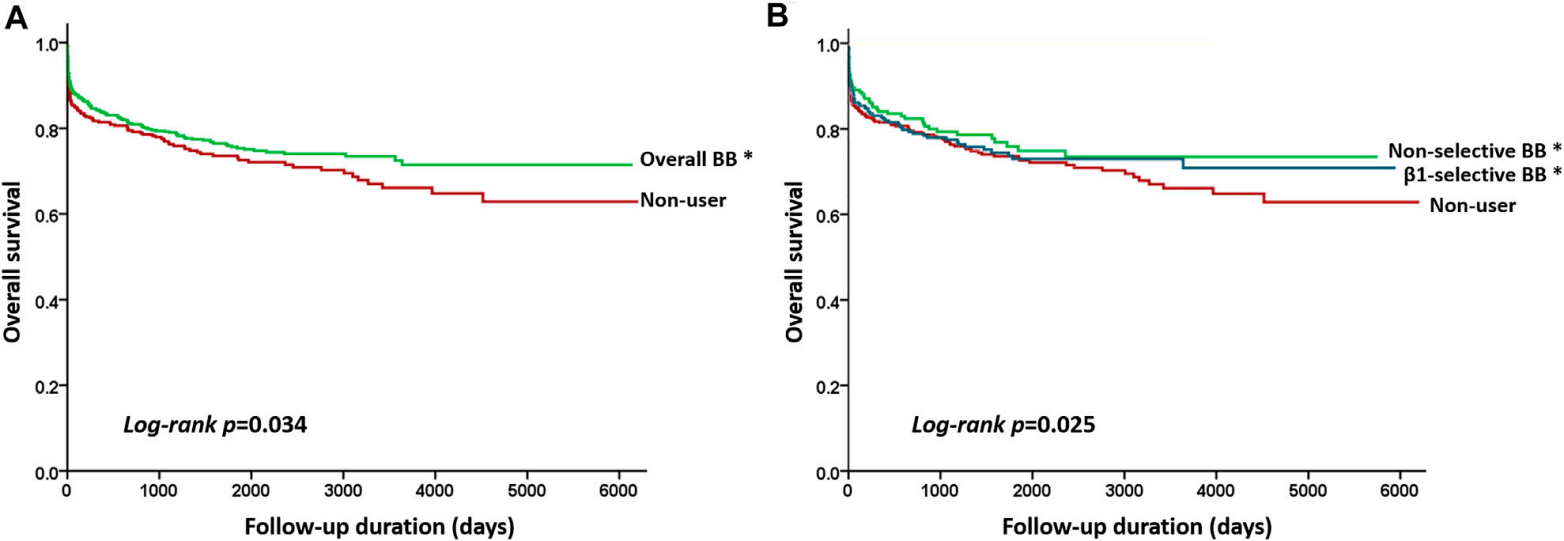
Kaplan–Meier curves showing the overall survival according to the different beta-blocker treatments. **(A)** Beta-blockers (green line) vs control (red line) (log-rant rest, *p* = 0.034), and **(B)** Kaplan–Meier curves by three treatment subgroups with pairwise comparisons using the log-rank test. * indicated *p* value <0.05 when compared with control. Statistically significant survival differences were noted between non-selective beta-blockers (green line) vs control group (red line) (log-rank test *p* = 0.027) and β1-selective beta-blockers (blue line) vs control group (log-rank test *p* = 0.036) but not between non-selective versus β1-selective beta-blockers (log-rank test *p* = 0.142).

**FIGURE 3:**
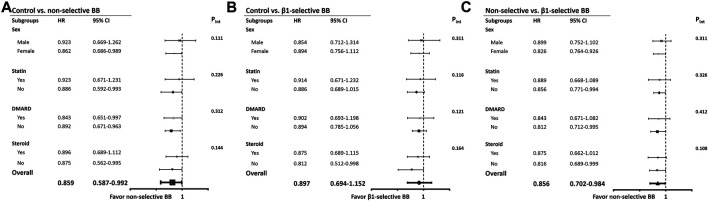
Subgroup analyses. **(A)** Hazard ratios of mortality in specific subgroups of ACEIs-treated patients by using controls as the reference group. **(B)** Hazard ratios of new-onset AF in specific subgroups of ARBs-treated patients by using controls as the reference group. **(C)** Hazard ratios of new-onset AF in specific subgroups of ACEIs/ARBs-treated patients by using controls as the reference group. Abbreviations: ACEIs, angiotensin converting enzyme inhibitors; ARB, angiotensin receptor blockers; HR, hazard ratio; CI, confidence interval; CVD, cardiovascular disease (combination of coronary artery disease, ischemic stroke, hemorrhagic stroke, peripheral artery disease); CHF, congestive heart failure; DM, diabetes mellitus; HTN, hypertension.

The results of Cox regression analyses are demonstrated in Table 3. Compared with patients not taking beta-blockers, the crude result showed that subjects using beta-blockers were significantly associated with a lower risk of all-cause mortality (model 1; HR, 0.841; 95% CI: 0.688–0.952). After adjusting for potential confounders, use of beta-blockers (model 2; adjusted HR, 0.871; 95% CI: 0.727–0.978) was associated with a lower risk of all-cause mortality. The relation between use of beta-blockers and the reduced risk of all-cause mortality remained significantly correlated after adjustment with propensity score (model 3; adjusted HR, 0.882; 95% CI: 0.724–0.982) (Table 3). We stratified the different treatment into three groups, including non-selective versus control, β1-selective beta-blockers versus control, and non-selective versus β1-selective beta-blockers. Compared with control, patients treated with non-selective beta-blockers had a significantly lower risk of mortality either in the crude analysis (model 1, HR 0.822 [95% CI: 0.638–0.915] and after adjustment (model 2, HR 0.759 [95% CI: 0.587–0.912], and model 3, HR 0.876 [95% CI: 0.760–0.975]. On the other hand, compared with control, patients treated with β1-selective beta-blockers had a significantly lower risk of mortality in the crude analysis (model 1, HR 0.879 [95% CI: 0.650–0.944]). However, the result did not remain significant after adjustment with cofounders (model 2, HR 0.847 [95% CI: 0.694–1.152], and model 3, HR 0.891 [95% CI: 0.710–1.154]). At last, we compared the risk of mortality between patients receiving non-selective and β1-selective beta-blockers. Patients with non-selective beta-blockers had a better prognosis than those with β1-selective beta-blockers (model 1, HR 0.884 [95% CI 0.712–0.916]; model 2, HR 0.856 [95% CI 0.702–0.984]; and model 3, HR 0.872 [95% CI 0.723–0.991]) (Table 3). To make sure our PH assumption was right, we conducted the plots of the scaled Schoenfeld residuals over time, which showed all the *p*-values of the Chi-square test were >0.05 (Supplementary Figure S1). The Kaplan-Meier survival curves are illustrated in Figure 2. We plotted survival curves based on the different treatment. The log-rank tests were significant in the beta-blocker vs control group (*p* = 0.034) (Figure 2A). In Figure 2B, the log-rank tests were reported by using pairwise comparison which were significant in the non-selective vs control group (*p* value = 0.027) and β1-selective beta-blockers vs control group (*p* = 0.036). However, the comparison was not significant between non-selective and β1-selective beta-blockers (*p* = 0.142). The results of subgroup analyses are demonstrated in Figure 3. Among the subgroups (sex and use of statins, DMARDs, and steroids), use of non-selective beta-blockers was associated with a lower risk of mortality when compared with control (Figure 3A). For patients treated withβ1-selective beta-blockers, the risk reduction was not significant among different subgroups (Figure 3B). However, when compared with use of β1-selective beta-blockers, subjects treated with non-selective beta-blockers had a better prognosis, irrespective of sex and medications (Figure 3C).

## Discussion

According to this nationwide cohort study, use of beta-blockers was associated with a lower risk of incidence of mortality in post-MI patients with RA, compared with no use of beta-blockers. In subgroup analysis, we found that treatment with non-selective beta-blockers was associated with an even lower risk of mortality in RA patients after MI, compared with use of *β*1-selective beta-blockers. The main results remained consistent after adjustment of the known confounders and propensity scores. To our best knowledge, this study is the first to evaluate the long-term efficacy of beta-blockers in patients with rheumatoid arthritis after myocardial infarction and discloses the better post-MI protection of non-selective beta-blockers in RA patients.

In the contemporary percutaneous coronary intervention (PCI) era, the overall mortality rate of post-MI patients treated with beta-blockers is approximately 3–4% over a 1 year follow-up in the general population (Ozasa et al., 2010; Nakatani et al., 2013; Choo et al., 2014). However, RA is associated with a more severe presentation of acute coronary syndrome and worse outcome (Mantel et al., 2015; Palomäki et al., 2021). In our study, the overall mortality rate was 5.71%, which was much higher than the general population. The risk of death was still higher despite of treatment with beta-blockers (non-selective and β1-selective beta-blockers: 4.64 and 5.79%, respectively). On the other hand, the mortality rate of the RA population after MI reported by Palomäki and his colleagues was 25.8% at 1 year and 51.0% at 5 years. In our cohort, the rate of treatment with beta-blockers after MI was around 70% and the overall mortality rate was 5.71% at 1 year, which was much lower than those reported by Palomäki et al. (2021). It might be explained by the rate of PCI/CABG during the index hospitalization. In our data, around 90% of patients received revascularization either by PCI or CABG, while there was around 40–45% who received revascularization in Palomäki’s cohort. The higher rate of revascularization in our cohort was because of the comprehensive NHI reimbursement and easy and friendly medical access in Taiwan. However, our data still presented the higher mortality rate and poor prognosis of RA patients after MI, which suggested the need of aggressive and comprehensive medical care in the RA population.

On the other hand, there was a sex difference in our cohort and more female patients received beta-blockers after MI. Indeed, sex disparities in cardioprotection of beta-blockers might be expected because estrogens inhibit the cardiac expression of β1-adrenoceptors and reduce β-adrenergic-mediated stimulation exerting cardioprotective effects (Kam et al., 2004; Kalibala et al., 2020). However, a meta-analysis recruiting 2,134 women with heart failure mostly due to MI presented a similar reduction in mortality in both sexes (Shekelle et al., 2003). Hypertension was more prevalent in the beta-blocker group, compared with control. The imbalance of the comorbidity of hypertension could explain why there were more female patients using beta-blockers, irrespective of non-selective and β1-selective in our analysis (Kalibala et al., 2020). A recent study suggested that beta-blocker use may be an acute precipitant of heart failure in new-onset coronary heart disease among women, but not men, which subsequently increased the risk of death (Bugiardini et al., 2020). However, our study demonstrated a better cardioprotection of non-selective beta-blockers, irrespective of men and women (Figure 3, subgroup analysis of sex).

The current guidelines recommend long-term beta-blockers therapy after MI, which reduces mortality and recurrent MI (Amsterdam et al., 2014; Ibanez et al., 2018). The protective effect remained consistent among patients with comorbidities, including diabetes, congestive heart failure, chronic obstructive pulmonary disease, and asthma (Gottlieb et al., 1998). There are survival benefits in mortality in both non-selective and *β*1-selective beta blockers. It is possible that beta-blockers exert a class effect (Lin et al., 2015). In the CAPRICORN trial, non-selective beta blocker, carvedilol, reduced all-cause and cardiovascular mortality (Dargie, 2001). On the other hand, *β*1-selective beta blocker, bisoprolol, also provided significant 2-year cardiac death and myocardial infarction reduction in high-risk patients (Poldermans et al., 2001). However, there were little data investigating the long-term effect of beta-blockers of post-MI patients with RA. Our study observed a reduced risk of mortality among RA patients treated with beta-blockers compared with non-users. In subgroup analysis, treatment with non-selective beta-blockers could provide more protection from death during long-term follow-up after MI. Rheumatoid arthritis is a disease characterized by chronic joint inflammation and bone destruction. Reactive oxygen species (ROS) play an important role in the pathogenesis of RA, and increasing ROS release is mainly related to TNF-alpha overproduction in patients with RA. Elevated ROS level causes tissue damage associated with inflammation (Mirshafiey and Mohsenzadegan, 2008). In an animal study, non-selective β-adrenoreceptor antagonist, propranolol, reduced oxidative stress and TNF-α signaling and demonstrated an anti-inflammation effect (Lin et al., 2020a). Several clinical trials observed the survival benefit of patients treated with propranolol in the following condition, such as severe burns, akathisia associated with Alzheimer’s disease, or psychosis and anxiety (Peskind et al., 2005; Ali et al., 2015). Among cancer patients, treatment with propranolol also showed a potential benefit on cancer recurrence and overall survival, by reducing inflammatory cytokines including IL-6 and TNF-alpha, inflammation-related transcription factors such as NFkB and STAT3, and reducing the activation of Treg lymphocytes (Zhou et al., 2016; Haldar et al., 2018). In addition, when COX-2 inhibitors were co-administrated with beta-blockers, there was a positive synergistic effect of anti-inflammation (Shaashua et al., 2017). Given the chronic inflammatory status of the RA population, the anti-inflammatory effect of non-selective beta-blockers could possibly explain the better survival benefit when compared with β1-selective beta-blockers.

In addition to an anti-inflammatory effect, potentially beneficial effects of beta-blockers in patients with MI include decreasing oxygen demand, improving diastolic function, reducing risk of ventricular arrhythmia, and balancing automaticity (Lopez-Sendon et al., 2004). Among these medications, statins could reduce CV events and mortality in RA patients in primary prevention but not in secondary prevention (Semb et al., 2011; Sheng et al., 2012; Danninger et al., 2014). In our study, the prevalence of statin usage in the β1-selective beta-blocker group was higher than other groups. However, non-selective beta-blockers were associated with a lower risk of mortality than β1-selective beta-blockers whether statin usage was adjusted or not. This finding was consistent with the results of the prior studies. There was no evidence of a synergistic effect with non-selective beta-blockers and statins in RA patients. Philip et al. reported the potential synergistic effect of beta-blockers and statins on overall mortality in patients after coronary artery bypass graft surgery (Philip et al., 2015). In their study population, there was no RA patients enrolled. On the other hand, use of DMARDs was associated with reduced risk of myocardial infarction and other cardiovascular events (Suissa et al., 2006; Greenberg et al., 2011). However, use of steroids may increase the risk of MI with a dose-depend effect (Aviña-Zubieta et al., 2012). These studies investigated the primary prevention effect of DMARDs and steroids in MI but the effect of secondary prevention was little investigated. Our subgroup analyses revealed that the secondary prevention effect of non-selective beta-blockers remained irrespective of use of statins, DMARDs, and steroids. Of note, the survival benefit was not significant in the subgroup taking β1-selective beta-blockers, which suggested the role of chronic inflammation in the RA population with MI (Sattar and Mcinnes, 2005; Solomon et al., 2006; Lin et al., 2020b). Furthermore, our results showed long-term survival improvement in the RA population, consistent with the general population.

### Limitation

There are a number of limitations in the present study. The most frequently prescribed beta-blocker after MI in other studies is metoprolol but the patient population using metoprolol in Taiwan was too small (only 8.1% of β1-selective beta-blockers). The mostly used beta-blockers in our cohort were propranolol, bisoprolol, and nebivolol. Secondly, we were unable to conduct a randomized, controlled trial; therefore, our results may have been affected by defects inherent to non-randomized comparisons. These include selection bias and an uneven distribution of risk factors. To address these issues, we conducted several statistical methods with utilization of propensity scores to control for detected differences between groups. On the other hand, it is possible that some factors were not properly accounted for. For example, we were unable to access data related to inflammatory biomarkers, seropositivity, or drug adherence, as this information is not available in the NHI database. Furthermore, rheumatoid arthritis is known to remarkably affect MI fatality. There were no data about creatinine kinase or troponin level to measure the infarct size and to investigate the relation between RA and MI. Finally, we did not adjust for in-hospital administration of beta-blockers; therefore, we are unable to evaluate the benefits of early beta-blocker usage after acute MI, which has demonstrated a better protective effect (Pizarro et al., 2014).

## Conclusion

The use of beta-blockers after MI improves prognosis in the general population. Among the RA population, in our nationwide cohort study, we observed a reduced risk of death among beta-blocker users compared with non-users, in particular those treated with non-selective beta-blockers. Our data provide evidence supporting the prescription of beta-blockers for RA patients after MI and further research is warranted to investigate the class effect of beta-blockers in the RA population.

## Data Availability

The raw data supporting the conclusion of this article will be made available by the authors, without undue reservation.

## Supplementary Material

Supplementary Figure 1 Plots of the scaled Schoenfeld residuals versus time for the Cox proportional hazards model fit to the entire dataset. The solid line gives the estimated effect of the predictors through time. Plots are given for **(A)** beta-blockers versus control, **(B)** non-selective beta-blockers versus control, **(C)** β1-selective beta-blockers versus control and **(D)** non-selective versus β1-selective beta-blockers. Abbreviations: BB, beta-blockers.
