# Supplementary figures and images for: Long-Term Effect of Non-Selective Beta-Blockers in Patients With Rheumatoid Arthritis After Myocardial Infarction—A Nationwide Cohort Study
Source: Front Pharmacol. 2021 Sep 21;12:726044. doi: 10.3389/fphar.2021.726044 (PMC8490958; doi:10.3389/fphar.2021.726044)

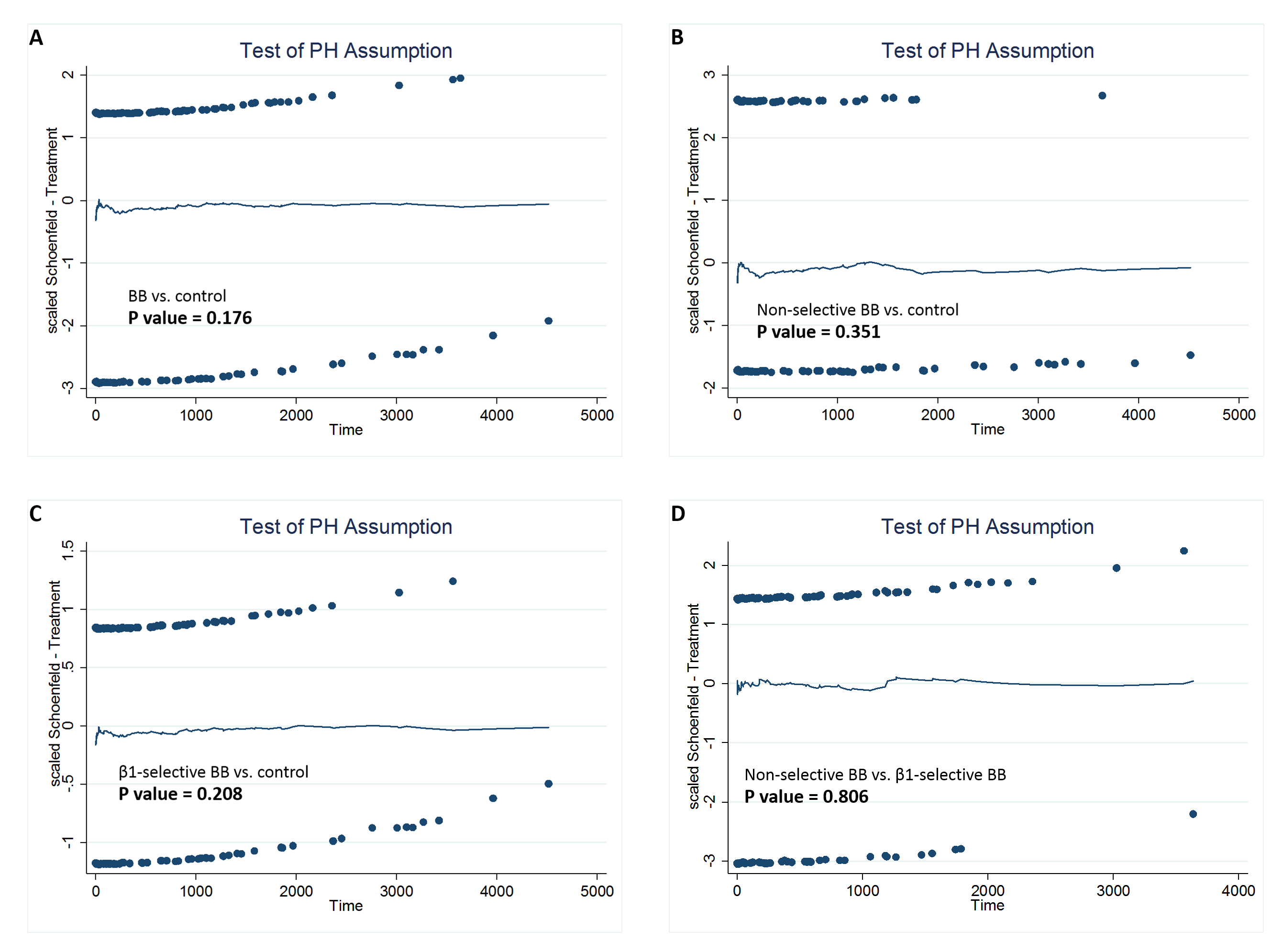

Supplement: Supplementary file 1 [file Image1.TIF]
